# Supplementary material for: Pegylated Liposomal Alendronate Biodistribution, Immune Modulation, and Tumor Growth Inhibition in a Murine Melanoma Model
Source: Biomolecules. 2023 Aug 26;13(9):1309. doi: 10.3390/biom13091309 (PMC10527549; doi:10.3390/biom13091309)
Supplement: Supplementary file 1 [file biomolecules-13-01309-s001.zip › biomolecules-2566369-supplementary.pdf]

Supplemental Materials

Manuscript Title: Pegylated Liposomal Alendronate Biodistribution, Immune modulation, and Tumor Growth Inhibition in a Murine Melanoma Model

Authors: Md. Rakibul Islam, Jalpa Patel, Patricia Ines Back, Hilary Shmeeda, Raja Reddy Kallem, Claire Shudde, Maciej Markiewksi, William C. Putnam, Alberto A. Gabizon, Ninh M. La-Beck

Table S1. Primer sequences for M1 and M2 macrophage markers.

Gene	Sequence
Ubc	F: 5′-CCAGTGTTACCACCAAGAAG-3′<br>R: 5′-ACCCAAGAACAAGCACAAGG-3′
Arg-1	F: 5′-ACCTGGCCTTTGTTGATGTCCCTA-3′<br>R: 5′-AGAGATGCTTCCAACTGCCAGACT-3′
iNOS	F: 5′-GTTCTCAGCCCAACAATACAAGA-3′<br>R: 5′-GTGGACGGGTCGATGTCAC-3′
IL-6	F: 5′-GCTACCAAACTGGATATAATCAGGAAA-3′<br>R: 5′-CTTGTTATCTTTTAAGTTGTTCTTCATGTACTC-3′
CXCL10	F: 5′-CCTATGGCCCTCATTCTCAC-3′<br>R: 5′-CTCATCCTGCTGGGTCTGAG-3′
IL-10	F: 5′-CGGGAAGACAATAACTGCACCC-3′<br>R: 5′-CGGTTAGCAGTATGTTGTCCAGC-3′
TGF-β	F: 5′-TGATACGCCTGAGTGGCTGTCT-3′<br>R: 5′-CACAAGAGCAGTGAGCGCTGAA-3′

Note: Ubc, ubiquitin C; Arg-1, arginase-1; iNOS, nitric oxide synthase; IL-6, interleukin-6; CXCL10, C-X-C motif chemokine ligand 10; IL-10, interleukin 10; TGF-β, transforming growth factor beta.

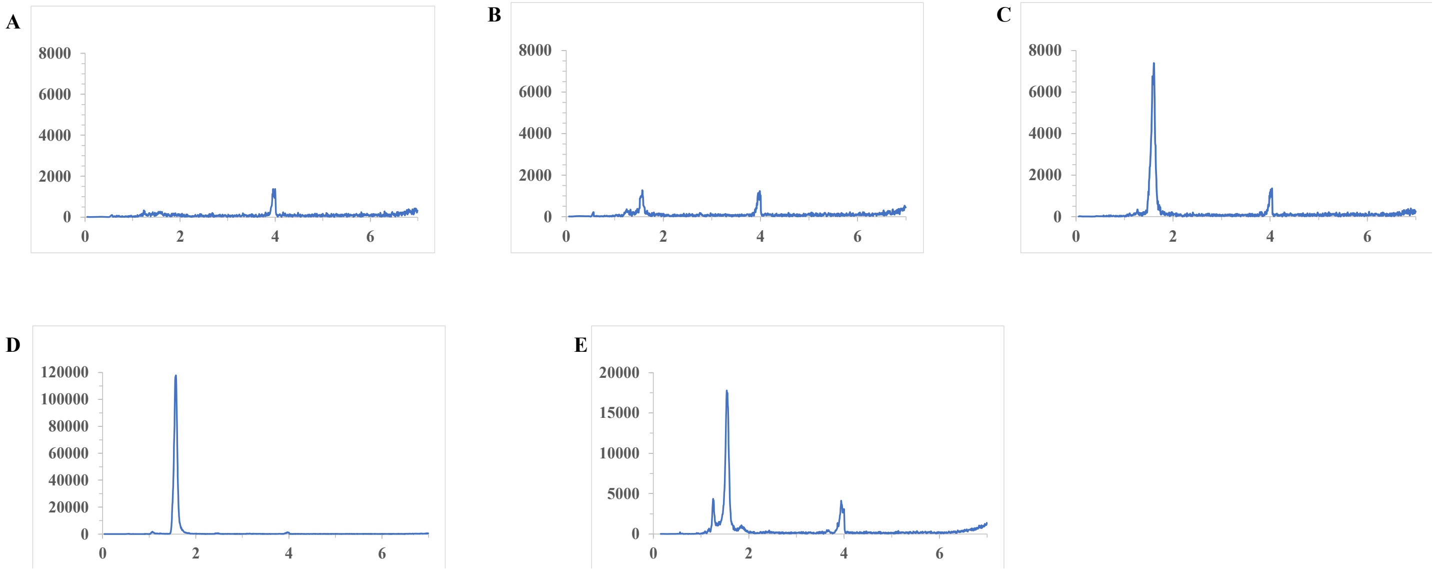

Figure S1. Representative chromatograms of alendronate in serum. (A) Serum Blank, (B) Serum standard (10ng/mL), (C) Serum QC (80ng/mL), (D) serum sample #1507, and (E) Internal standard: Alendronate-d6

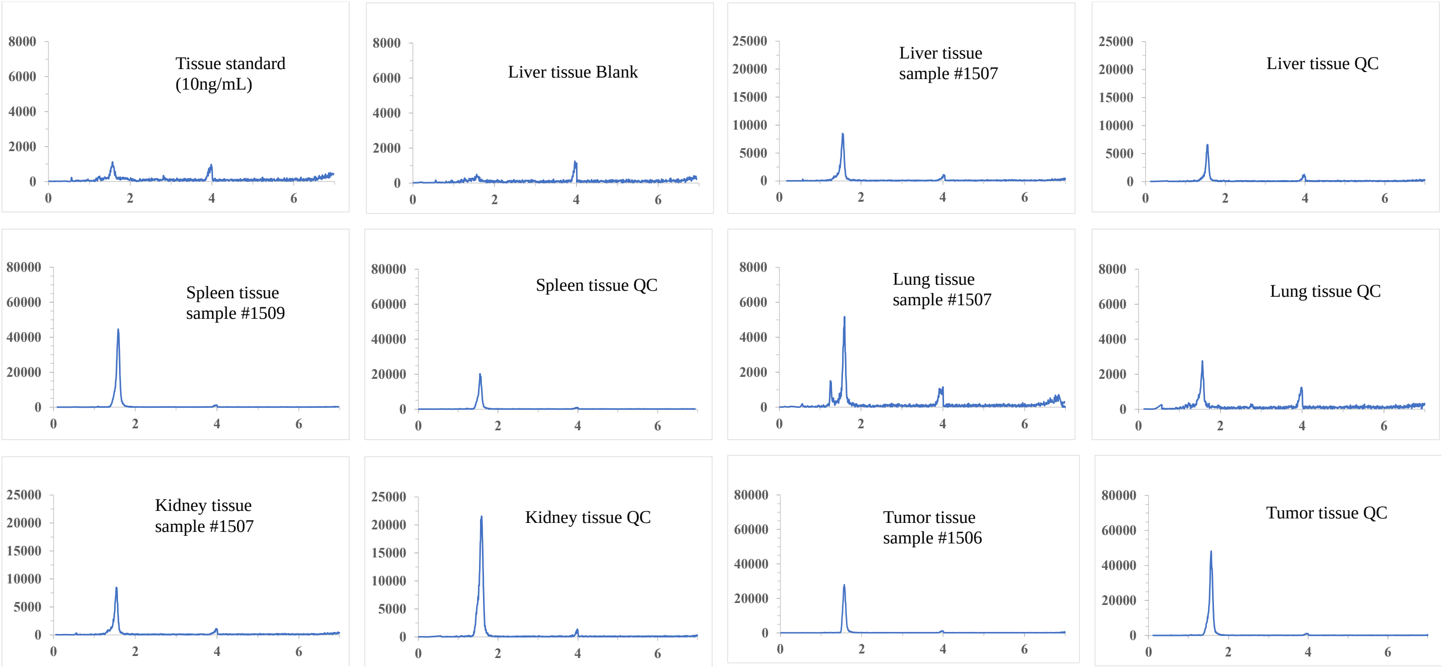

Figure S2. Representative chromatograms of alendronate in tissues.

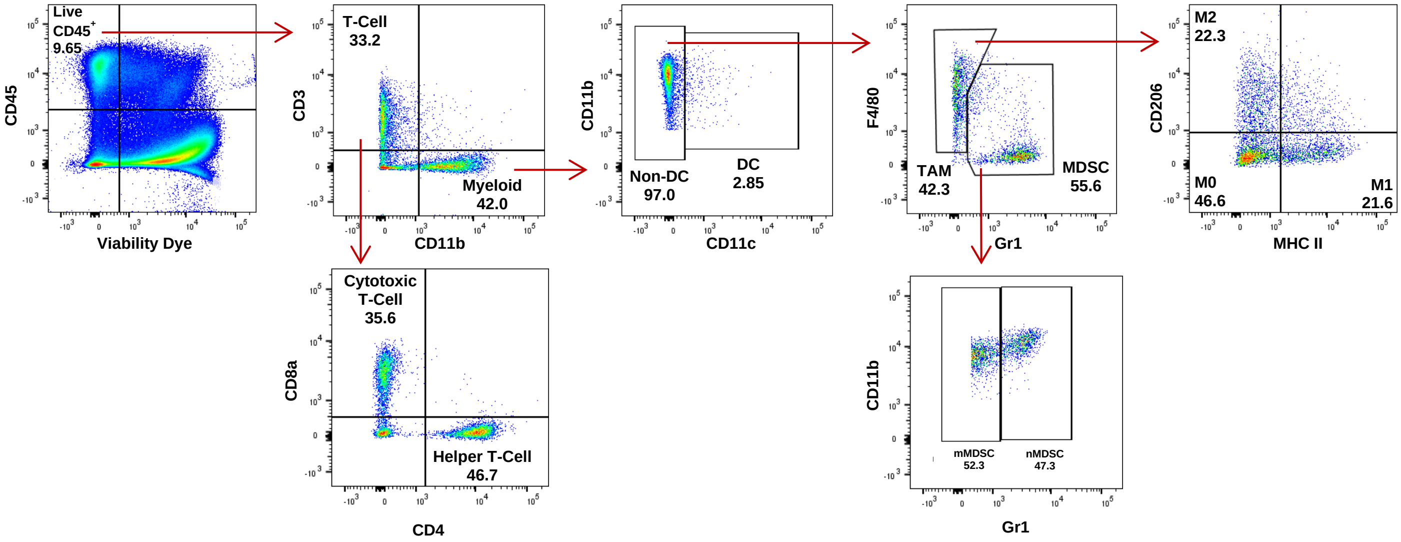

Figure S3. Gating strategy for immunophenotyping of B16-Ova tumor microenvironment.

Table S2: Characteristics of PLA formulations used in this study.

Liposomal Formulation	Size (nm)	PDI	pH	Phospholipid (mM)	Alendronate (mM)	Alendronate (mg/ml)
Batch A (PLA)	99.7	0.08	7.2	44.0	18.6	4.6
Batch B (PLA)	93.3	0.08	6.9	48.8	17.1	4.3
Batch C (PLA-Dil)	84.9	0.05	6.8	26.4	7.4	1.85

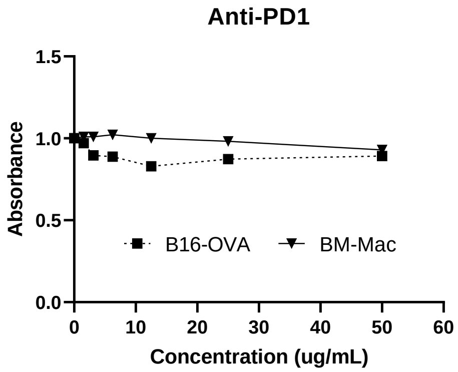

Figure S4. In vitro cytotoxicity of anti-PD1 therapy. Murine bone marrow-derived macrophages (Mac) and B16-OVA melanoma cells were treated with anti-PD1 mAb then cytotoxicity assessed using MTT assay. Viability is expressed as a ratio relative to untreated controls. Each treatment condition was performed in duplicate or triplicate, and at least two experimental replicates were conducted. Data represents mean from one experiment; coefficients of variation were <10% for all replicates.

Table S3. Treatment related mortality and morbidity in the B16-ova tumor model shows increased weight loss for PLA when compared to anti-PD1 and control group. Results are represented by number of mice with weight loss ≥ 10% or mortality per total mice in each treatment group.

Treatment	Weight loss ≥ 10%	Mortality
Vehicle + isotype	1/17	0/17
Vehicle + anti-PD1 (10 mg/kg)	1/19	1/19
PLA (4 mg/kg) + isotype	6/12	0/12
PLA (5 mg/kg) + isotype	6/6	0/6

Anti-PD1: anti-PD1 antibodies; PLA: PEGylated liposome alendronate.

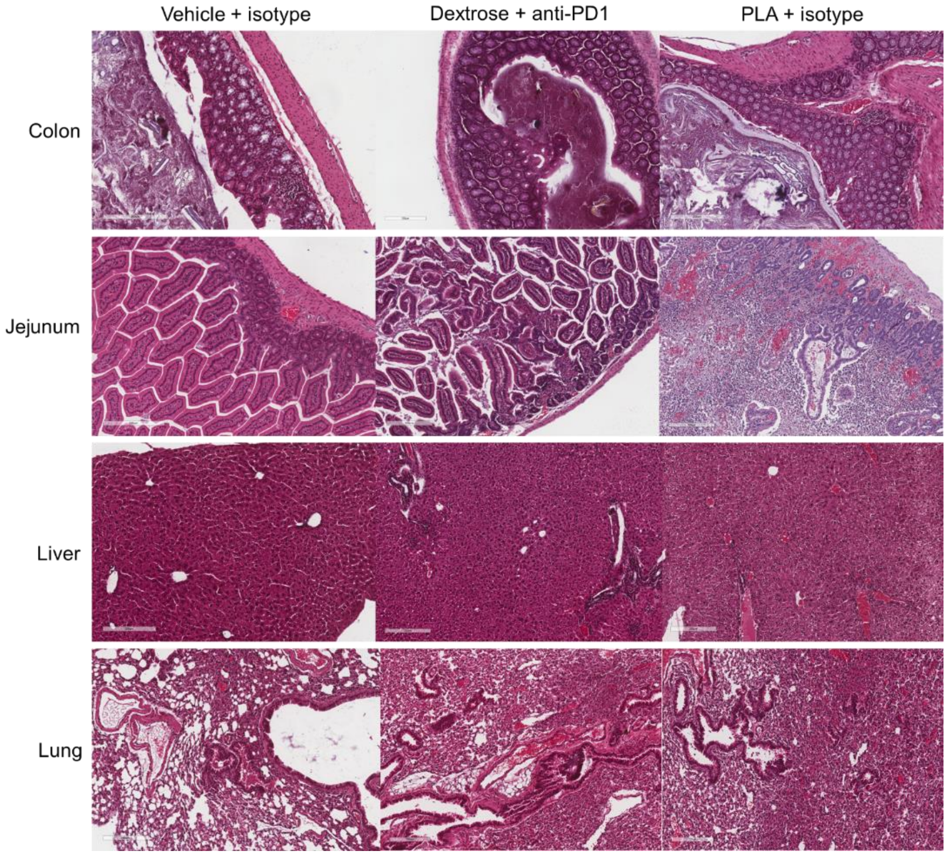

Figure S5. Histopathological analyses of treatment-related toxicity showing colon, jejunum, liver, and lung for control, anti-PD1, and PLA treated groups. Vehicle + isotype images represent inflammation baseline, while treatment groups images show highest level of inflammation encountered for each group.

Table S4. Histopathological analyses for each organ showing treatment-related toxicity from efficacy studies in the B16-OVA tumor model. Results represent the presence of inflammation per number of analyzed mice in each treatment group (A), and also broken down by grade of inflammation (B). Analyses were performed blinded to treatment identity by a certified pathologist (MM).

A) B16 ova tumor model.

Treatment	Colon	Jejunum	Stomach	Liver	Heart	Lung	Kidney	Spleen
Vehicle + isotype	3/3	1/3	0/2	1/3	0/3	2/3	0/3	0/2
Vehicle + anti-PD1 (10 mg/kg)	3/5	3/5	0/1	3/3	0/4	4/4	0/4	1/3
PLA (4 mg/kg) + isotype	2/3	4/5	1/1	0/3	0/3	2/3	0/3	1/2

αPD1: anti-PD1 antibodies; PLA: PEGylated liposome alendronate.

B) B16-ova tumor model inflammation grading.

Treatment	Colon		Jejunum		Stomach		Liver		Lung		Spleen	
	Any grade	High grade	Any grade	High grade	Any grade	High grade	Any grade	High grade	Any grade	High grade	Any grade	High grade
**Vehicle + isotype**	3	0	1	0	0	0	1	0	2	0	0	0
**Vehicle + anti-PD1** (10 mg/kg)	3	0	3	0	0	0	3	0	4	0	1	0
**PLA (4 mg/kg) + isotype**	3	0	3	1	1	0	0	0	1	0	1	0

Anti-PD1: anti-PD1 antibodies; PLA: PEGylated liposome alendronate.
